# Evaluating the Plantar Pressure Loading and Its Correlation to Craniocervical Posture in Subjects With Skeletal Class II Malocclusion Before and After Surgical Mandibular Advancement

**DOI:** 10.7759/cureus.48250

**Published:** 2023-11-04

**Authors:** Ratna Parameswaran, Radhika Srimagesh, Anantanarayanan Parameswaran, Devaki Vijayalakshmi R

**Affiliations:** 1 Orthodontics and Dentofacial Orthopaedics, Meenakshi Academy of Higher Education and Research, Chennai, IND; 2 Oral and Maxillofacial Surgery, Meenakshi Ammal Dental College, Chennai, IND

**Keywords:** occlusion, craniocervical posture, postural changes, bilateral sagittal split osteotomy, plantar pressure

## Abstract

Class II malocclusion is one of the most prevalent types of malocclusions following Class I. The most typical postural features observed in Class II patients are extended craniocervical posture, cervical lordosis, and increased forward loading away from the body’s centre of mass for compensating the mandibular deficiency and reduced airway. Its treatment involves orthodontic, functional, and orthognathic surgery. The type of treatment regimen opted for depends upon the age, severity of malocclusion, and need of the patient. Thus, appropriate intervention brings about improvement in dentition along with an overall enhancement of the body posture and plantar loading. These variables undergo a significant change whenever there is a change in the maxillomandibular relationship. The main aim of this paper is to correlate the variation in the craniocervical angulation to the plantar pressure distribution during various phases of orthodontic treatment and bilateral sagittal split osteotomy (BSSO) advancement surgery. The craniocervical angulations were determined from the lateral cephalograms of the patients using cephalometric variables and the plantar pressure was estimated using a digital quanpressurometer device (designed and patented in India by Meenakshi Academy of Higher Education and Research; patent number 390136) at each phase, i.e., during pre-treatment, pre-surgery, post-surgery, post-treatment intervals.

The outcome of this study indicated that there was a significant change in the craniocervical angulation and the plantar pressure distribution pattern of the subjects before and after orthognathic surgery and it remained constant for six months after orthognathic surgery. The limitation of the study was the limited sample size. This study reveals that there was an improvement in the craniocervical angulation and plantar pressure distribution during the course of orthodontic decompensation and orthognathic surgical treatment, thus bringing about change in the individual's overall body posture and their plantar loading pattern after orthognathic surgery. Therefore, the change in the stomatognathic system by orthodontic and orthognathic treatment influences the overall muscular and functional balance of an individual thereby improving their attitude and lifestyle.

## Introduction

The stomatognathic system is made up of a variety of structures connected to the head and spine to perform a variety of functions such as mastication, deglutition, speech, and respiration. This sophisticated system makes up a cybernetic regulatory environment interconnected with other systems, thereby maintaining the human body’s posture and orientation in space [1]. Generally, body posture can be elucidated as the orientation of the head and torso to gravity, the field of vision, and the point of support. Human balance is a dynamic process that remains consistent despite major and minor oscillations [2] whereas body posture has an extremely limited duration of oscillations, thereby forming a static movement. Orthodontic and orthognathic intervention brings about changes in the dental relationship as well as jaw bases thus these changes influence the fascia and the muscles invariably thereby bringing about changes in the body posture. This study mainly aims to highlight the changes in the craniocervical posture and the plantar pressure changes brought about by BSSO advancement in Class II adult patients.

Thus, dental occlusion and the stomatognathic system are invariably related to body posture and plantar pressure. Therefore, any alterations in body posture affect plantar pressure distribution. The dental system acts as a direct reflection of the incoordination between the pelvic rotation, thoracic, lordotic, and pelvic inclinations, and cervical spine posture [3]. The abnormal maxillomandibular relationship in Class II and Class III also influences the body posture by affecting the kinetic chain, where it primarily originates from changes in the temporomandibular joint articulation in case of retrognathia and prognathia, thereby producing changes in the masticatory muscle functions this change is further transmitted to the distal musculature via the muscle chain. When changes in the cervical musculature occur, it brings about alterations in the spinal column space provoking descending compensations in the lumbar and dorsal level. These compensations invariably bring about pelvic deviations’ and rotations, which bring about further changes in the limbs and foot pressure changes depending on the type of malocclusion.

Dental malocclusion has a profound impact on the postural control of an individual. Nobili et al. noted that, generally, individuals with Class II malocclusion majorly possess a protruded maxilla and retruded mandible [4]. This distocclusion results in forward positioning of the head and thereby the center of gravity of the body shifts forward seeking postural equilibrium. This also influences the plantar pressure distribution by shifting them anteriorly for compensating the mandibular deficiency [4]. Lombardo et al. stated that among the global prevalence of malocclusion, Class II was found to be 20.2% prevalent and it affects various age groups [5]. Generally, patients with skeletal Class II malocclusion possess a steep mandibular plane angle, extended craniocervical posture, and increased anterior facial height. Gresham et al. stated that sagittal deficiency of the mandible leads to more inferior positioning of C1, lordosis, and soft tissue stretching that impedes the further growth of the mandible [6]. Thus, this change in the trend of craniocervical posture occurs in concordance with previous studies in the literature, which concluded that the craniocervical posture also has an influence on malocclusion patterns. Extended head posture and craniocervical angulation were one of the causes of the development of Class II malocclusion [7].

Patients with Class II malocclusion have a deficient mandible associated with reduced airway and obstructive sleep apnea. Therefore, to compensate for the mandibular retrognathism and facilitate breathing, these patients possess more anterior tilting of the head and extension of the neck to improve the airway. This may develop as an inherent feature in Class II individuals during their growth. Okuro et al. stated that this change was established in 80.0% of individuals with skeletal Class II malocclusion [8]. Though there are different treatment strategies available for treating skeletal Class II malocclusion, orthognathic surgery is one of the standard methods of treating adult patients with severe Class II malocclusion. The primary goal of orthognathic surgery is to establish a proper maxillofacial relationship by relocating the facial skeletal structures and thus improving facial aesthetics and harmony. Several studies in the literature have highlighted the significance of orthodontic, functional, and orthognathic intervention of these malocclusions, which have a greater impact on the craniocervical angle and the overall body posture [9].

Orthognathic surgery is one effective method for correcting severe Class II malocclusion in adults. There can be an evident variation in the patient’s body posture and gait after surgery. This change is due to the change in the body’s center of mass to its intended position. Therefore, the body undergoes various postural changes during each phase of orthodontic treatment, before and after orthognathic surgery. Thus, establishing an appropriate vertical, sagittal posture and occlusal relationship invariably influences craniocervical posture and plantar pressure distribution, leading to a drastic improvement in the patient’s overall gait, sway, and body posture as a result of equalizing the pressure in the plantar region. The main scope of this study was to examine the improvement in the overall craniocervical angulations and plantar pressure distribution in subjects during pre-orthodontic, pre-surgical, post-surgical, and after a follow-up period of six months after orthodontic treatment.

## Case presentation

Materials and methods

The present study was conducted on patients who reported to the Department of Orthodontics, Meenakshi Ammal Dental College, Chennai. The study sample comprised five adult patients with skeletal Class II malocclusion seeking orthodontic treatment above the age of 18 years, These subjects were skeletal Class II patients indicative of Bilateral Sagittal Split Osteotomy (BSSO) advancement. The study sample comprised five adult patients with skeletal Class II malocclusion seeking orthodontic treatment above the age of 18 years. These patients were subjected to orthodontic treatment for decompensation for about six months followed by which orthognathic surgery was performed. The patients were followed up during the recovery phase. Therefore, during this period, these individuals were subjected to craniofacial and postural examinations. The patients involved in this study were indicative of undergoing BSSO advancement surgery. None of the subjects had previous orthodontic, orthopedic, or orthognathic treatment history, craniofacial abnormalities, temporomandibular joint (TMJ) disorders, bruxism, syndromes affecting gait and posture, or a history of preexisting spine problems.

The methodology for obtaining cephalograms and craniocervical angulations is as follows. Posture in humans can be of two types: it can be either by the visual righting system or crude positioning system which occurs with the help of proprioceptive muscle tendons and nerve impulses. The head posture in particular can be with external references (self-balance position) or without external references (mirror position). The main objective here was to obtain a lateral cephalogram of the patient’s cervical column as obtained by the patient’s own postural mechanism inherently; it is the most repeatable position and is determined as the orthostatic position of the patient [10]. The patients were made to walk on the spot before taking the radiograph, and then they were subjected to a lateral cephalometric evaluation. Thus, in this position, it is possible for us to easily observe the craniocervical angulations and tilt in body posture. Mirroring of the patient was done by asking the patient to look at their own eyes while taking the radiograph. The patient was positioned systematically by posture, for the feet, body, head, and asymmetry, in an orderly manner [11].

Radiographic variables

**Figure 1:**
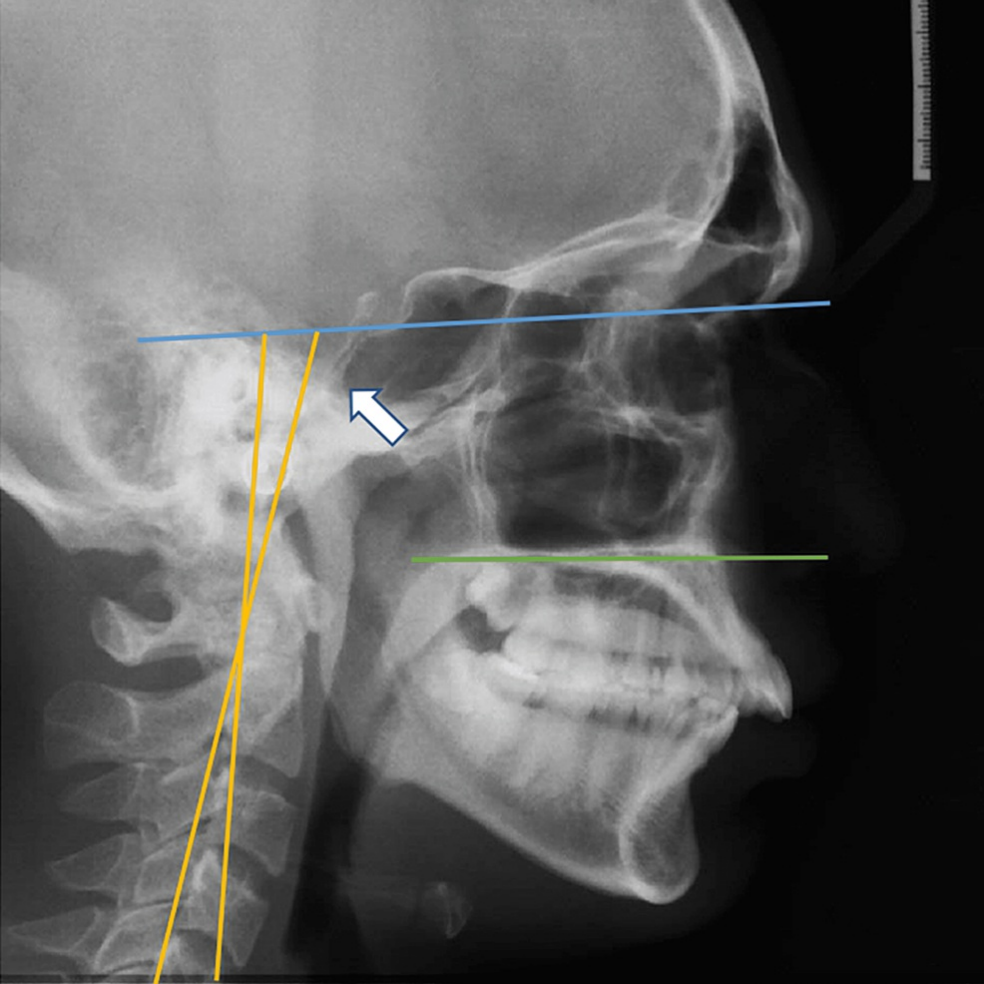
Pre-treatment lateral cephalogram of a skeletal Class II patient The image shows extended cervical posture – cervical lordosis with a convex profile. OPT/CVT - odontoid process tangent/cervical vertebra tangent; Blue line – Nasion-Sella (N-Se) line; Yellow line– indicates the OPT/CVT line; Green line – indicates maxillary plane; White arrow - OPT/CVT angle formed between the tangent to the cervical vertebrae and the tangent to the odontoid process.

Individuals were subjected to a lateral cephalometric evaluation at different intervals, that is, pre-treatment (T0), pre-surgical (T1), and post-treatment (T2). The craniocervical angulation was the first area of interest considered here. The odontoid process tangent/cervical vertebra tangent (OPT/CVT) angle is formed between the tangent to the cervical vertebrae and the tangent to the odontoid process. Thus, these values were obtained in the radiographs at all three phases of treatment. The pattern of craniocervical angle change was analyzed in all the subjects before and after BSSO advancement surgery. This angulation was used as a parameter for associating with the plantar pressure distribution to correlate the craniofacial skeleton with the overall body posture and loading conditions during decompensation periods and after the treatment. The pre-treatment (T0) radiographs were taken before the beginning of the treatment. This was taken in order to find the initial angulation and the postural adaptation of the cranium before decompensation (Figure 1).

**Figure 2:**
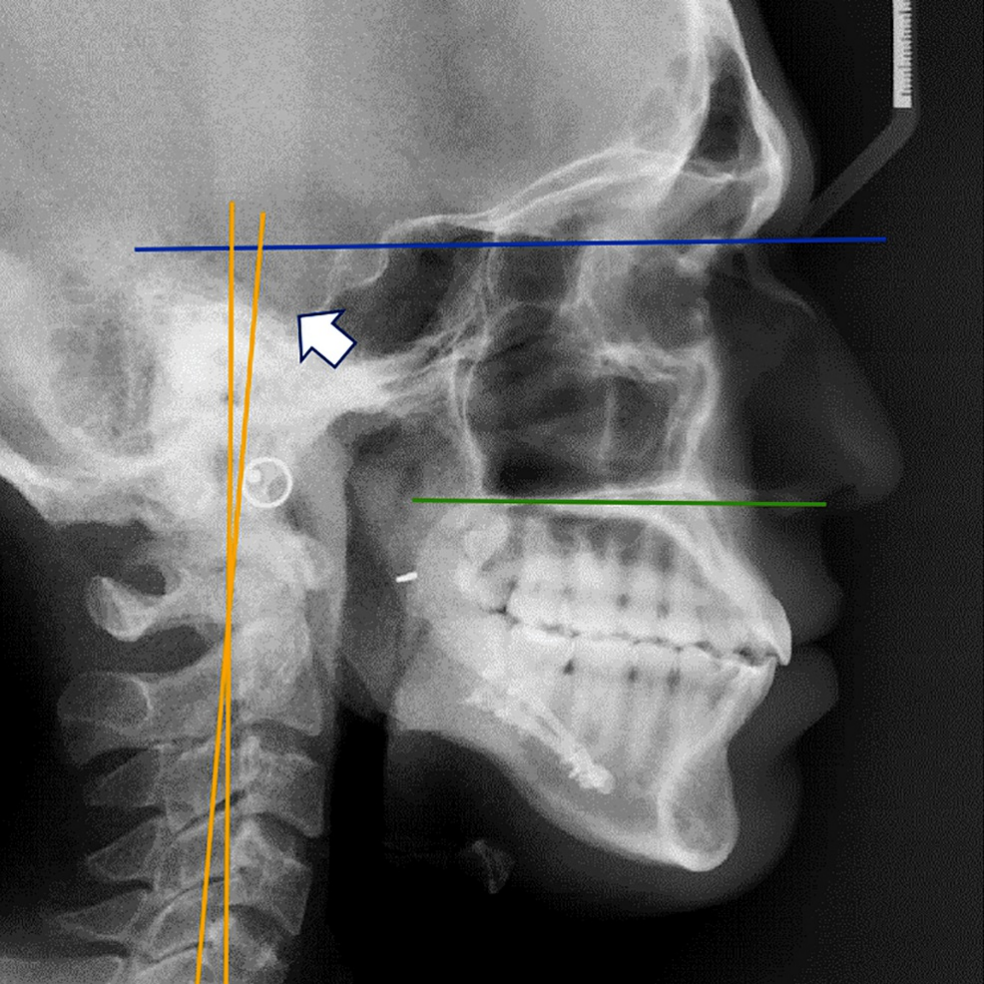
Post-treatment lateral cephalogram of the patient Lateral cephalogram after bilateral sagittal split osteotomy surgery of the patient revealing further up-righting of the craniocervical angulation and straightening of the profile. The image explains the uprighting of the craniocervical angulation compared to the presurgical phase (Figure 1). Blue line – Nasion-Sella (N-Se) line; Yellow line – indicates the OPT/CVT line; Green line – indicates maxillary plane; White arrow - OPT/CVT angle, which is formed between the tangent to the cervical vertebrae and the tangent to the odontoid process.

The pre-surgical (T1) radiograph was taken before the surgery. After the dental decompensation procedures, this was to determine whether the postural adaptation of the cranium improved or worsened after the commencement of the treatment. The post-treatment (T2) radiographs were taken after resolution of treatment i.e., six months after the surgery and commencement of orthodontic treatment (Figure 2).

Methodology for determining the plantar pressure

The plantar pressure distribution was evaluated using a custom-made digital quanpressurometer pressure platform device (designed and patented in India by Meenakshi Academy of Higher Education and Research; patent number 390136). The plantar pressure distribution was evaluated during pre-treatment (P0; before bonding), (P1; pre-surgical), post-surgical (P2; immediately after surgery), and post-treatment (P3; six months after surgery at debonding). The quanpressurometer device was used to determine the static loading and pressure of the individual. It comprised a foot platform, which consisted of sensors attached to the positions of different sizes from five to nine at United Kingdom standards. The platform consisted of sensors for each foot angulated at about 30 degrees to the midline with three pressure sensors at the foot of the following regions.

The variables used in the quanpressurometer device were medial calcaneus (P1; LP1-Left medial calcaneus; RP1-Right medial calcaneus), first metatarsal (P2; LP2-Left first metatarsal; RP2-Right first metatarsal), and hallux (P3; LP3-Left hallux; RP3-Right hallux). The strain gauge was constructed in such a way connecting the sensors for each foot on the platform and the strain gauge records the plantar pressure in the form of strain from each sensor. The device also comprised two vertical plurality rods on either end of the device and one horizontal plurality rod with multiple holes in it. Thus, threads can be inserted through these holes by forming a grid, making it easier to observe pelvic and shoulder tilts and vertebral inclination with respect to the body’s midline. The subjects were positioned on the pressure sensors according to the size of their feet, the readings were recorded at various periods of their treatment, and the plantar pressure distribution was evaluated.

Statistics

Descriptives

At pretreatment (R0), the mean pressure of RP1, RP2, RP3, LP1, LP2, and LP3 were 11+2.35, 23.4+9.81, 21.8+11.2, 13.2+4.87, 29.8+6.69, and 20.6+10.4, respectively. At post-treatment (R3), the mean pressure of RP1, RP2, RP3, LP1, LP2, and LP3 were 4.40+2.61, 5.4+2.70, 5.8+5.85, 4.8+2.68, 5+3.46, and 4.6+4.34, and respectively. This indicates that the pretreatment values of RP1, RP2, RP3, LP1, LP2, and LP3 have reduced considerably after treatment (Table 1). The mean pressure levels of all variables measured were found to be statistically significant with p-value < 0.05. One-way ANOVA was used to compare the means of all the variables measured (Table 2).

**Table 1:** Descriptive values of plantar pressure distribution LP1- Left medial calcaneus; RP1- Right medial calcaneus; LP2- Left first metatarsal; RP2- Right first metatarsal; LP3- Left hallux; RP3- Right hallux.

GROUP		RP1	RP2	RP3	LP1	LP2	LP3
Mean	Pre-treatment	11.0	23.4	21.8	13.2	29.8	20.6
	Post-treatment	4.40	5.40	5.80	4.80	5.00	4.60
	Pre-surgical	12.6	31.4	29.2	13.2	28.8	19.4
	Post-surgical	10.4	15.4	13.0	11.0	19.0	12.6
Median	Pre-treatment	12	25	19	10	34	27
	Post-treatment	3	5	4	5	3	4
	Pre-surgical	13	32	28	15	26	17
	Post-surgical	10	12	11	11	16	13
Standard deviation	Pre-treatment	2.35	9.81	11.2	4.87	6.69	10.4
	Post-treatment	2.61	2.70	5.85	2.68	3.46	4.34
	Pre-surgical	5.90	9.56	8.98	5.76	10.3	9.53
	Post-surgical	3.91	6.07	5.10	4.47	8.51	3.51

**Table 2:** One-Way ANOVA Welch’s test used *p-value<0.05 – statistically significant

GROUPS	F	df1	df2	P
Cervical angle	2.07	2	7.76	0.190
RP1	6.21	3	8.57	0.015*
RP2	14.95	3	7.77	0.001*
RP3	7.71	3	8.59	0.008*
LP1	5.48	3	8.48	0.022*
LP2	20.73	3	8.12	<0 .001*
LP3	5.75	3	8.32	0.020*

Tukey’s post-hoc test was used for multiple internal comparisons (Table 3-6). The reduction of mean pressure in RP1 from presurgical to post-treatment was found to be statistically significant with a p-value of 0.02. The reduction of the mean pressure in RP2 from pre-treatment to post-treatment was found to be highly statistically significant with a p-value of 0.009. The reduction of mean pressure in RP3 from pre-treatment to post-treatment was found to be statistically significant with a p-value of 0.03. The reduction of mean pressure in LP2 from pretreatment to post-treatment was found to be highly statistically significant with a p-value < 0.001.

**Table 3:** Multiple comparisons of RP1 between pre-treatment, post-treatment and post-surgical and multiple comparisons of RP2 between pre-treatment, post-treatment and post-surgical Post hoc Tukey test; *p-value<0.05: statistically significant

RP1		Pre-treatment	Post-treatment	Pre-surgical	Post-surgical
Pre-treatment	p-value	—	0.076	0.917	0.995
Post-treatment	p-value	0.076	—	0.022	0.117
Pre-surgical	p-value	0.917	0.022	—	0.815
Post-surgical	p-value	0.995	0.117	0.815	—
RP2		Pre-treatment	Post-treatment	Pre-surgical	Post-surgical
Pre-treatment	p-value	—	0.009	0.374	0.374
Post-treatment	p-value	0.009	—	< .001	0.202
Pre-surgical	p-value	0.374	< .001	—	0.020
Post-surgical	p-value	0.374	0.202	0.020	—

**Table 4:** Multiple comparisons of RP3 between pre-treatment, post-treatment and post-surgical Post hoc Tukey test; *p-value<0.05: statistically significant

RP3		Pre-treatment	Post-treatment	Pre-surgical	Post-surgical
Pre-treatment	p-value	—	0.031	0.497	0.353
Post-treatment	p-value	0.031	—	0.002	0.520
Pre-surgical	p-value	0.497	0.002	—	0.029
Post-surgical	p-value	0.353	0.520	0.029	—

**Table 5:** Multiple comparisons of LP1 and multiple comparisons of LP2 between pre-treatment, post-treatment, and post-surgical Post hoc Tukey test; *p-value<0.05: statistically significant

LP1		Pre-treatment	Post-treatment	Pre-surgical		Post-surgical
Pre-treatment	p-value	—	0.047	1.000	0.872	
Post-treatment	p-value	0.047	—	0.047	0.183	
Pre-surgical	p-value	1.000	0.047	—	0.872	
Post-surgical	p-value	0.872	0.183	0.872	—	

LP2		Pre-treatment	Post-treatment	Pre-surgical	Post-surgical
Pre-treatment	p-value	—	< .001	0.997	0.158
Post-treatment	p-value	< .001	—	< .001	0.997
Pre-surgical	p-value	0.997	< .001	—	0.221
Post-surgical	p-value	0.158	0.997	0.221	—

**Table 6:** Multiple comparisons of LP3 between pre-treatment, post-treatment, and post-surgical Post hoc Tukey test; *p-value<0.05- statistically significant

LP3		Pre-treatment	Post-treatment	Pre-surgical	Post-surgical
Pre-treatment	p-value	—	0.020	0.994	0.373
Post-treatment	p-value	0.020	—	0.033	0.373
Pre-surgical	p-value	0.994	0.033	—	0.508
Post-surgical	p-value	0.373	0.373	0.508	—

Correlation matrix

Spearman’s correlation test was used to find the correlation between each variable (Table 7). DRP2 was found to be highly correlated with DCA, and it was found to be statistically significant with a p-value of 0.04. Though DRP3 and DRP1 showed high correlation, with a correlation coefficient of 0.821, it was not statistically significant. Similarly, DLP2 and DCA showed high correlation without statistical significance. On the other hand, there was slight to moderate correlation between other variables as well, but they were not statistically significant.

**Table 7:** Correlation between DRP and DLP. Spearman’s correlation test; *p-value<0.05- statistically significant. DCA – Difference in Cervical Angle; DRP1 - Difference in Right medial calcaneus; DRP2 - Difference in Right first metatarsal; DRP3 – Difference in Right hallux; DLP1 - Difference in Left medial calcaneus; DLP2 - Difference in Left first metatarsal; LP3 - Difference in Left hallux.

Spearman’s Correlation Matrix		DCA	DRP1	DRP2	DRP3	DLP1	DLP2	DLP3
DCA	correlation	—	-0.344	-0.894	-0.671	0.224	-0.803	-0.344
	p-value	—	0.571	0.041	0.215	0.718	-0.803	0.571
DRP1	correlation	-0.344	—	0.462	0.821	0.205	0.553	0.605
	p-value	0.571	—	0.434	0.089	0.741	0.334	0.279
DRP2	correlation	-0.894	0.462	—	0.700	-0.100	0.975	0.718
	p-value	0.041	0.434	—	0.233	0.950	0.005	0.172
DRP3	correlation	-0.671	0.821	0.700	—	0.400	0.667	0.564
	p-value	0.215	0.089	0.233	—	0.517	0.219	0.322
DLP1	correlation	0.224	0.205	-0.100	0.400	—	-0.154	0.205
	p-value	0.718	0.741	0.950	0.517	—	0.805	0.741
DLP2	correlation	-0.803	0.553	0.975	0.667	-0.154	—	0.816
	p-value	0.102	0.334	0.005	0.219	0.805	—	0.092
DLP3	Correlation	-0.344	0.605	0.718	0.564	0.205	0.816	—
	p-value	0.571	0.279	0.172	0.322	0.741	0.092	—

## Discussion

Various studies in the literature have discussed the correlation between dental malocclusion and its influence on body posture. According to Michalakis et al. [12] it is suggested that there is a relationship between dental malocclusion and the craniocaudal and podal systems. Therefore, malocclusion inadvertently influences body posture, plantar pressure, and foot-ground relationship [13]. The foot is used for balance and movement of an individual. Thus, any changes in the stomatognathic system are mirrored in the plantar pressure distribution [14]. This could be further explained as a change in the soft tissue stretch of the patient i.e. the “Soft Tissue Stretch” hypothesis after orthognathic surgery. The change in the mandibular position post-treatment brings about a gradual change in the craniocervical column whereby uprighting them. Since in Class II individuals, the center of gravity is positioned more anteriorly as a compensatory mechanism for deficient mandible post surgically this forward plantar loading decreases thereby correcting the body’s posture, implying that orthodontic and orthognathic treatment brings about overall changes in the dentofacial relationship and body posture.

Thus, orthodontic and orthognathic intervention to address dental and skeletal abnormalities inadvertently influences the individual's postural system thereby bringing about an evident improvement in them. Therefore, in this study, the plantar pressure distribution was evaluated at all stages of orthodontic treatment, and the results reveal that the plantar pressure was increased anteriorly in the RP1, RP2, LP1, and LP2 regions compared to the RP3 and LP3 ones, that is, in the posterior region. This was in concordance with the study conducted by Sandoval et al. [15].

Cuccia and Caradonna [16] stated that in Class II malocclusion, there is increased forward posturing of the head and vertebral column. As a result, the center of gravity of the body shifts anteriorly because an interconnected change produces increased forward loading. Therefore, when these individuals were subjected to orthognathic surgery, it was observed that there was an exaggeration of plantar loading in the anterior region during the decompensation phase pre surgically and the anterior plantar pressure loading decreased evidently after orthognathic BSSO advancement surgery post surgically. This change in the pattern of plantar pressure loading is because of the equalization of the pressure in both the anterior and posterior regions of the foot post-surgically. The equalization of plantar pressure positively related to decreased craniocervical angulations during the postoperative periods. This was in line with Chung et al. [17] who stated that the changes associated with the change in posture after orthognathic surgery are due to the stretching of the infrahyoid and the suprahyoid muscles, which causes further change in the posture of the vertebral column and the entire body balance as explained by Santos et al. [18].

There was a significant realignment in the skeletal posture that lasted for only three months after mandibular advancement surgeries in Class II patients after orthognathic surgery [19]. Generally, the postural changes that are brought about by orthodontic intervention last only for a shorter duration; this is because orthodontic treatment brings about only transient changes in the soft tissues and it does not influence the skeletal relationship of the individual this is the reason why the craniocervical changes are not so evident in orthodontic treatment. When orthodontic treatment is combined with orthognathic treatment, they bring about a long-term change in the body posture by alteration in the kinetic muscular chain and the podal system. Thus, our results were in contradiction to this study. Study subjects saw an improvement in static body posture. This was evident from the plantar pressure values and the craniocervical posture values, which significantly improved and were maintained even after six months of BSSO advancement surgery. The plantar pressure was equalized in the hallux, metatarsi, and calcaneus after orthognathic surgery, and it was also maintained for about six months after orthognathic surgery without any relapse. This preservation in the plantar pressure distribution for a prolonged period could have been due to the versatile realignment of the craniofacial musculature and the podal system to orthognathic intervention. The limitation of this research was that it was conducted only on a limited number of subjects since it was performed for an extended duration in orthognathic surgical patients. Therefore, extended research is required with a larger number of subjects.

## Conclusions

Orthodontic and Orthognathic treatment involves the rehabilitation of dentofacial deformities and malocclusion. Thus, an appropriate treatment strategy brings about improvement in body posture and gait. In Class II malocclusion there is a deficient mandible; on postural examination, it shows an increased posterior plantar loading pressure that could have been because of the alteration in the natural head position change and the change in the craniocervical angulations during the pre- and post-treatment periods.

The DRP2 and DLP2 were correlated with craniocervical angulations which were suggestive of an evident alteration in the lateral loading of the plantar region as the craniocervical angle was uprighting. The plantar pressure distribution followed a pattern of increase in RP1, RP2, RP3, LP1, LP2, and LP3 during the decompensation phase i.e., the pre-surgical phase and it decreased during the post-surgical phase this change was gradual and was sustainable after six months of post-surgical period. Thus, among the plantar pressure variables the anterior loading in RP1, RP2, LP1, and LP2 more evidently reduced during the post-treatment period which was suggestive of a decrease in the plantar pressure loading in the anterior region; this could have been due to the change in the kinetic chain caused as a result of forward repositioning of the mandible. This equalization of the plantar pressure was evident in the results obtained where Spearman’s correlation revealed a statistically significant change in the craniocervical uprighting and the plantar pressure equalization thereby shifting the body’s center of gravity from anterior to posterior (centering the center of gravity of the body) uprighting the gait.

Orthodontic camouflage treatment in adults involves the masking of an underlying skeletal discrepancy. Although this treatment modality is opted for by many patients compared to surgical interventions like BSSO advancement and genioplasty, this modality brings about changes only in the dental component of an individual; therefore, they do not influence the longstanding inherent posture and gait. When orthodontic treatment is combined with orthognathic surgical treatment to address skeletal discrepancies along with dental malocclusion, they act as a one-stop, wholesome treatment modality to bring about changes in facial esthetics, body posture, and gait.

In this research on evaluating the relationship between plantar pressure and craniocervical angulation in adult patients before and after orthognathic surgery, it was evident that there was a significant increase in the craniocervical posture and the plantar pressure distribution during the decompensation phases of orthodontic treatment. The plantar pressure and the craniocervical angulation decreased evidently after BSSO advancement surgery and the decrease in the cervical posture and the plantar pressure in the lateral region were more statistically significant and were persistent after orthodontic and orthognathic intervention.

## References

[1] Bergamini M, Pierleoni F, Gizdulich A, Bergamini C (2008). Dental occlusion and body posture: a surface EMG study. Cranio.

[2] Roggia B, Santos VA Filha, Correa B, Rossi ÂG (2016). Posture and body balance of schoolchildren aged 8 to 12 years with and without oral breathing (Article in English, Portuguese). Codas.

[3] Lippold C, Danesh G, Schilgen M, Drerup B, Hackenberg L (2006). Relationship between thoracic, lordotic, and pelvic inclination and craniofacial morphology in adults. Angle Orthod.

[4] Nobili A, Adversi R (1996). Relationship between posture and occlusion: a clinical and experimental investigation. Cranio.

[5] Lombardo G, Vena F, Negri P (2020). Worldwide prevalence of malocclusion in the different stages of dentition: a systematic review and meta-analysis. Eur J Paediatr Dent.

[6] Solow B, Kreiborg S (1977). Soft-tissue stretching: a possible control factor in craniofacial morphogenesis. Scand J Dent Res.

[7] Rocabado M, Johnston BE Jr, Blakney MG (1982). Physical therapy and dentistry: an overview. J Craniomandibular Pract.

[8] Okuro RT, Morcillo AM, Sakano E, Schivinski CI, Ribeiro MÂ, Ribeiro JD (2011). Exercise capacity, respiratory mechanics and posture in mouth breathers. Braz J Otorhinolaryngol.

[9] Souki MQ (2016). Severe Angle Class III skeletal malocclusion associated to mandibular prognathism: orthodontic-surgical treatment. Dental Press J Orthod.

[10] Sandham A (1988). Repeatability of head posture recordings from lateral cephalometric radiographs. Br J Orthod.

[11] Siersbaek-Nielsen S, Solow B (1982). Intra- and interexaminer variability in head posture recorded by dental auxiliaries. Am J Orthod.

[12] Michalakis KX, Kamalakidis SN, Pissiotis AL, Hirayama H (2019). The effect of clenching and occlusal instability on body weight distribution, assessed by a postural platform. Biomed Res Int.

[13] Michelotti A, Buonocore G, Manzo P, Pellegrino G, Farella M (2011). Dental occlusion and posture: an overview. Prog Orthod.

[14] Scharnweber B, Adjami F, Schuster G, Kopp S, Natrup J, Erbe C, Ohlendorf D (2017). Influence of dental occlusion on postural control and plantar pressure distribution. Cranio.

[15] Sandoval C, Díaz A, Manríquez G (2021). Relationship between craniocervical posture and skeletal class: a statistical multivariate approach for studying Class II and Class III malocclusions. Cranio.

[16] Cuccia A, Caradonna C (2009). The relationship between the stomatognathic system and body posture. Clinics (Sao Paulo).

[17] Chung DH, Hatch JP, Dolce C, Van Sickels JE, Bays RA, Rugh JD (2001). Positional change of the hyoid bone after bilateral sagittal split osteotomy with rigid and wire fixation. Am J Orthod Dentofacial Orthop.

[18] Santos JG, Montezuma T, Perez CS, Sverzut CE, Trivellato AE, Guirro EC (2021). Body postural realignment in the first 2 months after orthognathic surgery. Am J Orthod Dentofacial Orthop.

[19] Lin X, Edwards SP (2017). Changes in natural head position in response to mandibular advancement. Br J Oral Maxillofac Surg.

